# Molecular Epidemiology of Carbapenem-Resistant *Acinetobacter baumannii* Isolates from Northern Africa and the Middle East

**DOI:** 10.3390/antibiotics10030291

**Published:** 2021-03-11

**Authors:** Paul G. Higgins, Ralf Matthias Hagen, Bernd Kreikemeyer, Philipp Warnke, Andreas Podbielski, Hagen Frickmann, Ulrike Loderstädt

**Affiliations:** 1 Institute for Medical Microbiology, Immunology, and Hygiene, University of Cologne, 50935 Cologne, Germany; paul.higgins@uni-koeln.de; 2 German Centre for Infection Research (DZIF), Partner Site Bonn-Cologne, 50935 Cologne, Germany; 3 Department of Microbiology and Hospital Hygiene, Bundeswehr Central Hospital Koblenz, 56070 Koblenz, Germany; ralfmatthiashagen@bundeswehr.org; 4 Institute for Medical Microbiology, Virology and Hygiene, University Medicine Rostock, 18057 Rostock, Germany; bernd.kreikemeyer@med.uni-rostock.de (B.K.); philipp.warnke@med.uni-rostock.de (P.W.); andreas.podbielski@med.uni-rostock.de (A.P.); or frickmann@bnitm.de (H.F.); 5 Department of Microbiology and Hospital Hygiene, Bundeswehr Hospital Hamburg, 20359 Hamburg, Germany; 6 Department of Hospital Hygiene & Infectious Diseases, University Medicine Goettingen, 37075 Göttingen, Germany

**Keywords:** *Acinetobacter baumannii*, war injury, Libya, Syria, Iraq, Afghanistan, epidemiology, sequence typing, carbapenem resistance, resistance gene, crisis zone

## Abstract

At the Bundeswehr Hospitals of Hamburg and Westerstede, patients repatriated from subtropical war and crisis zones of Northern Africa and the Middle East were medically treated, including microbiological assessment. Within a six-year interval, 16 *Acinetobacter* spp. strains, including 14 *Acinetobacter baumannii* (Ab) isolates with resistance against carbapenems and origins in Afghanistan (*n* = 4), Iraq (*n* = 2), Libya (*n* = 2), and Syria (*n* = 8) were collected. While clonal relationships of Libyan and Syrian strains had been assessed by superficial next generation sequencing (NGS) and “DiversiLab” repetitive elements sequence-based (rep-)PCR so far, this study provides core genome-based sequence typing and thus more detailed epidemiological information. In detail, sequencing allowed a definitive species identification and comparison with international outbreak-associated Ab strains by core genome multi locus sequence typing (cgMLST) and the identification of MLST lineages, as well as the identification of known resistance genes. The sequence analysis allowed for the confirmation of outbreak-associated clonal clusters among the Syrian and Afghan Ab isolates, indicating likely transmission events. The identified acquired carbapenem resistance genes comprised *bla*_OXA-23_, *bla*_OXA-58_, *bla*_NDM-1_, and *bla*_GES-11_, next to other intrinsic and acquired, partly mobile resistance-associated genes. Eleven out of 14 Ab isolates clustered with the previously described international clonal lineages IC1 (4 Afghan strains), IC2 (6 Syrian strains), and IC7 (1 Syrian strain). Identified Pasteur sequence types of the 14 Ab strains comprised ST2 (Syrian), ST25 (Libyan), ST32 (Iraqi), ST81 (Afghan), ST85 (Libyan), and ST1112 (Syrian), respectively. In conclusion, the study revealed a broad spectrum of resistance genes in Ab isolated from war-injured patients from Northern Africa and the Middle East, thereby broadening the scarcely available data on locally abundant clonal lineages and resistance mechanisms.

## 1. Introduction

During the last two decades, military conflicts have affected many regions of Northern Africa, the Middle East and neighboring countries, including Afghanistan, Iraq, Libya, and Syria. As previously summarized [1,2], war-associated wounds are prone to colonization or infection with multidrug-resistant Gram-negative bacteria including *Acinetobacter baumannii* (Ab).

The epidemiology of war trauma-associated infections, nosocomial transmission as well as deployment-associated colonization with *A. baumannii* has been best studied during the recent military interventions of US-American and British Forces in Iraq [3,4,5,6,7,8,9,10,11,12,13]. Epidemiological data also exist from civilian Iraqi health care facilities [14,15] as well as from the field of environmental biology [16]. As early as in 2006, *bla*_OXA-58-like_ and *bla*_PER-like_ genes were identified via sequencing sequenced in Iraqi *A. baumannii* strains [4]. Six years later, identified carbapenemase genes comprised of *bla*_OXA-40_, *bla*_OXA-23_, *bla*_OXA-58_, and IS*Abal* located upstream of the intrinsic *bla*_OXA-51_ on plasmids and/or the chromosome [10]. In addition, *bla*_OXA-40-like_ genes were detected in *Acinetobacter* strains isolated in the Iraqi Kurdistan region. In 2008, multidrug-resistant European clone II-associated isolates were described to be linked with local outbreaks in soldiers deployed to Iraq [6]. From the Iraqi Kurdistan region, the Pasteur sequence types [17] ST2, ST136, ST94, ST623, ST792, and ST793 were reported. Although skin colonization had been discussed as a predictor of later traumatic *A. baumannii*-associated wound infections [5], a considerable proportion of 27% infections did not show respective clonal links [8]. Interestingly, there was even a decline in *A. baumannii* colonization in US troops during post-deployment screening from 21% to 4% between 2005 and 2009 [9].

Similar experience with multidrug resistant *A. baumannii* strains leading to trauma-associated wound infections were reported from the military conflicts and civil-war-haunted settings in Afghanistan [4,7,9,18,19,20,21], Syria [22,23,24,25,26], and Libya [27,28,29,30,31,32]. While *bla*_NDM-1_ was identified in carbapenem-resistant *A. baumannii* strains from Syria [23,24], alternative beta-lactam resistance mechanisms like *bla*_OXA-94_ (a *bla*_OXA-51_ variant) as well as the elements *ISAba13*, *ISAba14*, and *ISAba17* were also detected in strains of the ST85 clone [26]. In addition, *gyrA*-gene- and *parC*-gene-based resistance to fluoroquinolones was reported from those strains isolated from Syrian civil war victims [26]. In Libyan patients, *A. baumannii* strains carrying the carbapenemase-encoding gens *bla*_NDM-1_, *bla*_OXA-23-like_, and *bla*_OXA-40like_ were detected as well as high abundance of sequence type ST2 [27,29,30].

In the course of a six-year-interval, 16 *Acinetobacter* spp. isolates with reduced susceptibility towards carbapenems and origins in Afghanistan (*n* = 4), Iraq (*n* = 2), Libya (*n* = 2), and Syria (*n* = 8) were collected at the Bundeswehr Hospitals Hamburg and Westerstede, Germany. At those sites, patients repatriated from subtropical war and crisis zones of Northern Africa and the Middle East were medically treated and assessed. Superficial epidemiological and resistance information about the Libyan and Syrian strains had previously been provided by low-coverage next generation sequencing (NGS) and “DiversiLab” repetitive elements sequence-based (rep-)PCR [33], however, a detailed analysis of clonal relationship and resistance mechanisms was still missing.

In order to provide this information, whole genome sequencing as well as analysis based on core genome multi locus sequence typing (cgMLST) [34] were performed in the present study. By performing these analyses, we aimed at providing additional, more detailed epidemiological information on the international distribution of resistant *Acinetobacter* clones, as well as on underlying molecular resistance mechanisms.

## 2. Results

### 2.1. Confirmation on Species Level and Clustering with International Outbreak Strains Based on Core Genome Analysis

**Figure 1:**
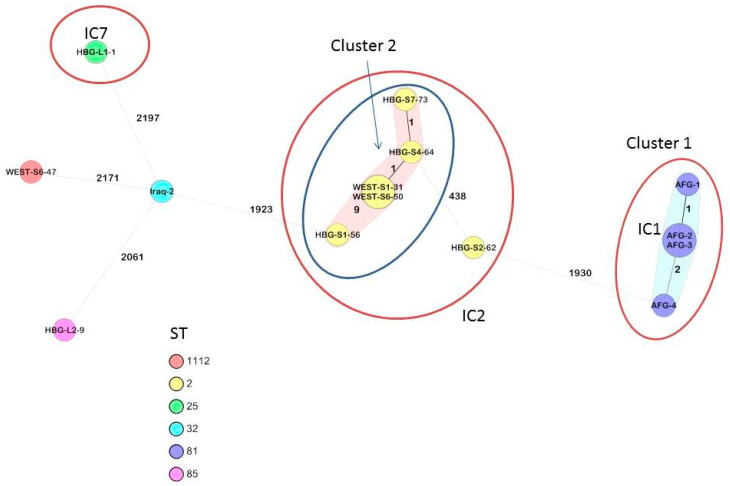
Minimum spanning tree of the *A. baumannii* strains based on 2390 target alleles (core genome). Isolate numbers are within the nodes, and the numbers between the nodes indicate the number of alleles that were different. Isolates are colored based on their Pasteur sequence type.

From the 16 *Acinetobacter* spp. isolates assessed, 14 were confirmed as *A. baumannii* by the *gyrB* multiplex PCR and presence of *bla*_OXA-51-like_. The remaining two strains were identified as *A. dijkshooriniae* (sample name Iraq-1) and *A. radioresistens* (sample name WEST-S5-44) using the Jspecies Tetra correlation search [35] and matrix-assisted laser-desorption-ionization time-of-flight mass spectrometry (MALDI TOF MS) (Table 1). Details on the sequence-based identification of these two *Acinetobacter* spp. on species level are provided in the Appendix A Table A1. All 14 *A. baumannii* isolates were subjected to cgMLST analysis, which showed that the isolates clustered with the previously identified international clonal lineages IC1 (the four Afghan strains), IC2 (six out of seven Syrian strains), IC7 (one out of two Libyan strains) [36], as shown in Figure 1, while for three isolates (one from Iraq, one from Syria, one from Libya), no matching IC could be identified.

Of note, cgMLST (Figure 1) confirmed the close clonal relationship of the four IC1 strains which had been isolated in the course of a small local outbreak in Afghanistan, suggesting a common source of infection. Regarding the Syrian *A. baumannii* strains of the IC2 cluster comprising isolates from two different Bundeswehr Hospitals (WEST for “Bundeswehr Hospital Westerstede” and HBG for “Bundeswehr Hospital Hamburg”), the clonal relationship that had already been suggested by rep-PCR and superficial sequencing (for comparison, also see Figure 2 in reference [33]) could be confirmed by cgMLST, making transmission events prior to hospital admission likely.

All isolates were typed based on two 7-loci multi-locus sequence typing (MLST) schemes, i.e., the Oxford scheme and the Pasteur scheme (Table 1) [37,38]. Notably, six of the *A. baumannii* were Pasteur ST-2 which corresponds to IC2, but were subdivided into four Oxford STs, while four isolates were Pasteur ST-81 (Oxford ST-498) which is a single locus variant of ST-1 and corresponds to IC1. The other isolates, including the non-*A. baumannii*, were represented by single STs.

### 2.2. Identified Molecular Resistance Mechanisms and Comparison with Phenotypic Resistance Testing

Table 1 summarizes the analysis of antimicrobial resistance determinants, ordered by strain, MLST type and international clonal lineage. Identified molecular resistance determinants of the carbapenem-susceptible Iraqi *A. dijkshooriniae* strain and the Syrian *A. radioresistens* strain are shown as well.

In most cases, isolates that were related carried the same resistomes. The most frequently detected resistance mechanisms among the assessed isolates comprised beta-lactamases as well as sulfonamide and aminoglycoside resistance genes. This is reflected by observed phenotypic resistance as indicated in the Appendix A Table A2, indicating high resistance rates against these substance groups, in particular for the isolates WEST-S6-47 and HBG-S2-62 which were resistant to amikacin, gentamicin, and tobramycin. These two isolates also possessed the same array of aminoglycoside modifying enzymes, as well as being the only isolates with *dfrA7, aac(6′)Ib-cr-like*, *cmlA1-like*, and *bla*_GES-11_, suggesting a shared plasmid or resistance island (Table 1). All *A. baumannii* were carbapenem resistant and this was mediated through possession of *bla*_OXA-23_ in the Afghan strains, by *bla*_OXA-23_ and/or *bla*_GES-11_ in the Syrian *isolates*, by *bla*_OXA-58_ in the Iraqi isolate, and *bla*_NDM-1_ in the Libyan isolate. Fluoroquinolone resistance was observed for all *A. baumannii* isolates (Appendix A Table A2). Analysis of the *gyrA* and *parC* genes revealed that the resistant isolates had a Ser83-Leu substitution in GyrA, while all but one (Iraq-2) also harbored a Ser80-Leu substitution in ParC. Resistance determinants to sulfonamides were present in all isolates resistant to co-trimoxazole despite a *dfr* gene being present in only two isolates, suggesting other mechanisms such as efflux might be playing a role, while *tetB-like* genes were detected in four isolates. All isolates remained susceptible to colistin.

All *A. baumannii* were in possession of the intrinsic *bla*_OXA-51-like,_ the variants of which correlated with their Pasteur STs and IC clonal designation (Table 1). IS*Aba1* was not associated with this gene. The *A. dijkshoorniae* possessed *bla*_OXA-819_, which is similar to the instrinic *bla*_OXA-213-like_ from the closely related *A. pittii*, while the *A. radioresistens* had *bla*_OXA-815_ which is a *bla*_OXA-23-like_ that is intrinsic to this species. Furthermore, all *A. baumannii* isolates carried their intrinsic *bla*_ADC_.

## 3. Discussion

The assessment was conducted to expand the knowledge about the micro-epidemiology of *A. baumannii* isolates with resistance against carbapenems from patients from Northern Africa and the Middle East, thus extending the results of a previous study published in 2018 [33]. A total of 16 *Acinetobacter* spp. strains including 14 *A. baumannii* from the strain collection of the Department of Microbiology and Hospital Hygiene, Bundeswehr Hospital Hamburg, were included in the study. Therefore, in addition to the previous study, clinical isolates from patients from Afghanistan and Iraq, which had been first introduced in a technical evaluation scheme on diagnostic identification of *Acinetobacter* spp. by fluorescence in situ hybridization (FISH) [39], could be included in the analysis. In comparison to the previous epidemiological assessment based on rep-PCR and superficial sequencing [33], cgMLST- and MLST-based typing applying the Pasteur and Oxford schemes [37,38] provided considerably more details, allowing origin-specific assignments to international clones and MLST-based sequence types. The comparably small size of the collection of those rare strains made a complete assessment of all available isolates, even of the non-*A. baumannii* strains, possible without any economic restrictions. Of note, all assessed *A. baumannii* isolates could be assigned to MLST sequence types of the Oxford and Pasteur schemes [37,38].

The cgMLST approach confirmed two nosocomial transmission clusters. Cluster 1 comprised the four Afghan isolates, which were isolated during a small local outbreak, while five out of seven Syrian *A. baumannii* strains which were isolated in 2013 at the Bundeswehr Hospitals Westerstede and Hamburg formed cluster 2. The remaining five isolates were singletons. The latter clustering as observed with the cgMLST-typing confirmed likely transmission events between Syrian patients prior to admission at the Bundeswehr Hospitals Westerstede and Hamburg, which had already been suspected based on the results of rep-PCR and superficial sequencing [33]. Those transmission events might either have occurred in medical facilities in the patients′ home country but also, as previously discussed for Syrian and Ukrainian war-injured patients treated at German military hospitals [33,40,41], during the evacuation flights out of the patients’ crisis-struck home countries. Of note, two strains isolated from Syrian patients at the military hospital in Westerstede (“WEST”-strains) showed completely identical sequences and other isolates of this cluster, which were isolated at the Bundeswehr Hospital Hamburg (“HBG”-strains) showed only one, two, and nine allele differences, respectively (Figure 1). Such very close genetic relationship makes recent transmission events highly likely.

When comparing the cluster 2 from Figure 1 of the results chapter with the epidemiology at the Bundeswehr hospitals as recently described [33], the resulting likely nosocomial transmission events were as follows. All patients had been admitted to the German Bundeswehr Hospitals at the same day in 2013. Focusing on the Bundeswehr Hospital Westerstede, the strain WEST-S1-31, which had been isolated at day 21 after admission from the anus of a patient, was most likely nosocomially acquired from another patient, from which strain WEST-S6-50 had been isolated at day 1 after admission from a wound. Focusing on the cluster-related strains HBG-S1-56, HBG-S4-64 and HBG-S7-73 isolated at the Bundeswehr Hospital Hamburg, the first two isolates had already been isolated at day 2 after admission from a deep wound and the perineal skin of the patients, respectively, so nosocomial transmission had most likely occurred prior to hospital admission. The strain HBG-S7-73, however, had been isolated at day 9 from the pharynx of a patient. The sequence homology as indicated in Figure 1 makes nosocomial transmission from the patient carrying the strain HBG-S4-64 at the perineal skin highly likely.

As an interesting side-effect, cgMLST confirmed the clonal relationships as suggested by rep-PCR in the recent assessment [33], thus additionally confirming the basic reliability of this comparably cheap and easy-to-perform typing approach for local outbreak scenarios, in spite of the undeniably higher resolution of genome-based typing [34,40,42]. A comparison with other *A. baumannii* isolates from Ukrainian conflict zones that we have investigated did not show any clonality to those in the current study (data not shown) [40].

When looking at the detected beta-lactam resistance genes, the quantitatively most relevant one was *bla*_OXA-23_, which could be identified in strains from all assessed geographic regions, and which is also frequent in resistant *A. baumannii* in Europe and beyond [43]. Other beta-lactamase genes with relevance for the observed carbapenem resistance comprised *bla*_OXA-58_, the class A beta-lactamase gene *bla*_GES-11_, and *bla*_NDM-1_, respectively. It is interesting to note that isolate WEST-S6-47 had the lowest carbapenem-MICs of the *A. baumannii* in this study and it only had the acquired *bla*_GES-11._ In addition, and especially when looking for the resistome beyond just beta-lactamases, considerable diversity could be seen even in spite of an otherwise low number of allelic differences between some isolates within the observed clonal clusters, which is suggestive for the abundance of plasmids and mobile genetic elements. Despite the high-level of resistance seen in these isolates, colistin still retained activity.

Any comparisons with previous reports from the geographic regions of interest are limited by a paucity of data from respective war and crisis zones. Further, the relatively low number of available isolates is, of course, insufficient to provide a comprehensive overview on locally abundant resistant *A. baumannii* clones as well as their molecular resistance determinants, an admitted limitation of the study. Nevertheless, the *bla*_OXA-58_ gene as detected in the Iraqi *A. baumannii* strain is one of the genes mediating carbapenem resistance which had been reported already in 2006 and thus early after the beginning of the local conflict [4]. In a similar way, the occurrence of *bla*_NDM-1_ and *bla*_OXA-23_ in *A. baumannii* isolated in Libya had been described before [27,29,30], making those detections not further surprising. In so far, several of the reported findings are not completely new but just complement previously available epidemiological information.

## 4. Materials and Methods

### 4.1. Patient Isolates

All *Acinetobacter* strains with reduced susceptibility towards carbapenems and origin from North African and Middle Eastern war and crisis zones available at the laboratory of the Bundeswehr Hospital Hamburg were included in the assessment. There were no exclusion criteria. In detail, the strains Iraq-1 and Iraq-2 were isolated from wounds of injured US-American soldiers in the Iraqi conflict and kindly provided by the US Landstuhl Regional Medical Center (LRMC) as described elsewhere [39]. The strains AFG-1, AFG-2, AFG-3, and AFG-4 isolated from casualties of the Afghanistan conflict were shipped via the German field hospital in Mazar-e Sharif as previously detailed as well [39]. No patient specific data were provided for those strains for security reasons. The origin of the strains from patients injured in the conflicts in Libya and Syria and treated at the Bundeswehr Hospitals Hamburg and Westerstede (HBG-L1 (1), HBG-L2 (9) from Libya; WEST-S1 (31), WEST-S5 (44), WEST-S6 (47), WEST-S6 (50), HBG-S1 (56), HBG-S2 (62), HBG-S4 (64), HBG-S7 (73) from Syria) was described in detail elsewhere [33] next to epidemiological links and a preliminary molecular resistance analysis based on superficial NGS and rep-PCR typing. No environmental isolates were available. *A. baumannii* were identified using MALDI-TOF-MS and *gyrB* multiplex PCR [44,45].

### 4.2. DNA Extraction and Whole Genome Sequencing

DNA extraction and whole genome sequencing was performed exactly as described recently [40]. In detail, MagAttract HMW DNA Kits (Qiagen, Hilden, Germany) were used to extract the genomic DNA of the isolates according to the manufacturer’s instructions for whole genome sequencing (WGS). Nextera XT library prep kits (Illumina GmbH, Munich, Germany) were applied for the preparation of sequencing libraries for 250 bp paired-end sequencing runs on an Illumina MiSeq sequencer. The Velvet assembler integrated in the Ridom SeqSphere+ v.7.0.4. software allowed the de novo assembling of the obtained reads. Any raw sequencing reads from the project were submitted to the European Nucleotide Archive (https://www.ebi.ac.uk/ena/, last accessed on 10 March 2021) with the Accession numbers PRJEB42650.

### 4.3. Molecular Epidemiology and Determination of Antibiotic Resistance Genes

All isolates were initially screened for the presence of common carbapenemases by PCR [46,47]. Using the pubMLST website (https://pubmlst.org/abaumannii/, last accessed on 10 March 2021)), the genome assemblies of all isolates were used to identify sequence types (STs) according to the Oxford and the Pasteur 7-loci MLST schemes [17,37,38].

Based on a validated core genome multi-locus sequence typing (cgMLST) scheme [34] using the Ridom SeqSphere^+^ v. 7.0.4 software (Ridom GmbH, Münster, Germany), the isolates were further analyzed. The clonal relatedness was visualized using the same software by generating a minimum spanning tree including 2390 target alleles, also including an in-house library with the established international clones.

Finally, the beta-lactamase database (http://www.bldb.eu/, last accessed on 10 March 2021)) as well as the webtool Resfinder [48,49] were applied for the identification of resistance genes.

### 4.4. Phenotypic Resistance Testing and Spectrometry-Based Discrimination

A preliminary discrimination of the strains to the *A baumannii* complex (ABC) level had been performed applying MALDI TOF MS as described previously [33]. In detail, a Shimadzu/Kratos “AXIMA Assurance” MALDI TOF MS (Shimadzu Deutschland GmbH, Duisburg, Germany) and the “IVD-mode VitekMS-ID” database version 3.2.0.-6 (bioMérieux, Marcy-l′Étoile, France) had been used. Regarding phenotypic resistance testing, colistin susceptibility was assessed applying the microbroth dilution assay MICRONAUT-S (MERLIN Diagnostika GmbH, Bornheim, Germany). All other tested substances were assessed applying AST-N248 cards in a VITEK-II automated device (bioMérieux).

### 4.5. Ethical Clearance

Ethical clearance for the molecular typing of the *Acinetobacter* strains was obtained from the ethics committee of the medical association of Hamburg (WF-042/15) in line with National German laws, without the need for informed consent.

## 5. Conclusions

In conclusion, this assessment provided epidemiological information on carbapenem-resistant *A. baumannii* strains as isolated from patients from Northern African and Middle Eastern war and crisis zones and deposited at the Bundeswehr Hospital of Hamburg, Germany. Respective epidemiological assessments, further analyzing the molecular background of the distribution and spread of antimicrobial resistance in such difficult-to-access regions in the world, are neglected so far, as technological resources for molecular surveillance purposes are scarce in politically unstable areas. Certainly, our current small assessment of more or less randomly isolated strains collected over several years cannot replace continuous surveillance programs but may provide a glimpse on information which is otherwise difficult to obtain and usually relies on small cross-sectional assessments [1,2,3,4,5,6,7,8,9,10,11,12,13,14,15,16,17,18,19,20,21,22,23,24,25,26,27,28,29,30]. Considering the ongoing problem of infections due to carbapenem resistant *A. baumannii* isolates worldwide, the provided data may serve as a piece in the puzzle of global antimicrobial resistance without diminishing the need for larger future studies on the local epidemiology of carbapenem resistance in Northern African and Middle Eastern *A. baumannii* isolates in order to amend and broaden this preliminary information.

## Figures and Tables

**Table 1:** Analysis of antimicrobial resistance determinants, ordered by strain, MLST type, and the international clonal lineage of the assessed *Acinetobacter* spp. isolates. N.a. = not applicable, ST = Sequence type, IC = International clone. Clusters are indicated in the same colors as in Figure 1.

Sample, Species, Country of Origin, Year of Isolation	MLST		Clonal Lineage		Antibiotic Resistance Determinants						
	STox	STpas		Sulfonamide	Phenicol	Beta-Lactam	Aminoglycoside	Macrolide	Tetracycline	Trimethoprim	Fluoroquinolone and Aminoglycoside
Iraq-2, *A. baumannii*, Iraq, 2010	1627	32	n.a.	*sul2*-like		*bla*_ADC-25-like_, *bla*_OXA-100_, *bla*_OXA-58_	*aadB*-like				
AFG-1, *A. baumannii*, Afghanistan, 2008	498	81	IC1	*sul2*		*bla*_ADC-25-like_, *bla*_OXA-23_, *bla*_OXA-69_	*aadB-*like, *aph(3′)-Ia*				
AFG-2, *A. baumannii*, Afghanistan, 2008	498	81	IC1	*sul2*		*bla*_ADC-25-like_, *bla*_OXA-23_, *bla*_OXA-69_	*aadB-*like, *aph(3′)-Ia*				
AFG-3, *A. baumannii*, Afghanistan, 2008	498	81	IC1	*sul2*		*bla*_ADC-25-like_, *bla*_OXA-23_, *bla*_OXA-69_	*aadB-*like, *aph(3′)-Ia, aph(3′)-VIa-*like				
AFG-4, *A. baumannii*, Afghanistan, 2008	498	81	IC1	*sul2*		*bla*_ADC-25-like_, *bla*_OXA-23_, *bla*_OXA-69_	*aadB-*like, *aph(3′)-Ia*				
HBG-L1-1, *A. baumannii*, Libya, 2011	440	25	IC7	*sul2*		*bla*_ADC-25-like_, *bla*_OXA-23_, *bla*_OXA-64_	*aadB-*like, *aph(3′)-Ic, strA, strB*				
HBG-L2-9, *A. baumannii*, Libya, 2011	1089	85	n.a.	*sul2*	*floR*-like	*bla*_ADC-25-like,_*bla*_NDM-1_, *bla*_OXA-94_	*aadB-*like, *aph(3′)-VIa-*like	*mph(E), msr(E)*			
WEST-S6-47, *A. baumannii*, Syria, 2013	2271	1112	n.a.	*sul1*	*cmlA1*-like	*bla*_ADC-25-like_, *bla*_GES-11_, *bla*_OXA-715_	*aadA2, aadB, aph(3′)-VIa-*like, *strA-*like, *strB-*like, *aacA4-*like			*dfrA7*	*aac(6′)Ib-cr*-like
WEST-S1-31, *A. baumannii*, Syria, 2013	218	2	IC2	*sul2*		*bla*_ADC-25-like_, *bla*_OXA-23_, *bla*_OXA-66_, *bla*_TEM-1D_	*aph(3′)-Ic, armA,* *strA, strB*	*mph(E), msr(E)*	*tet(B)*-like		
WEST-S6-50, *A. baumannii*, Syria, 2013	218	2	IC2	*sul2*		*bla*_ADC-25-like_, *bla*_OXA-23_, *bla*_OXA-66_, *bla*_TEM-1D_	*aph(3′)-Ic, armA,* *strA, strB*	*mph(E), msr(E)*	*tet(B)*-like		
HBG-S4-64, *A. baumannii*, Syria, 2013	218	2	IC2			*bla*_ADC-25-like_, *bla*_OXA-23_, *bla*_OXA-66_, *bla*_TEM-1D_	*aph(3′)-Ic, armA,* *strA, strB*	*mph(E), msr(E)*	*tet(B)*-like		
HBG-S1-56, *A. baumannii*, Syria, 2013	195	2	IC2			*bla*_ADC-25-like_, *bla*_OXA-23_, *bla*_OXA-66_, *bla*_TEM-1D_	*aph(3′)-Ic,* *strA, strB*		*tet(B)*-like		
HBG-S7-73, *A. baumannii*, Syria, 2013	218	2	IC2			*bla*_ADC-25-like_, *bla*_OXA-23_, *bla*_OXA-66_, *bla*_TEM-1D_	*aph(3′)-Ic, armA,* *strA, strB*	*mph(E), msr(E)*	*tet(B)*-like		
HBG-S2-62, *A. baumannii*, Syria, 2013	1114	2	IC2	*sul1,sul2*	*cmlA1*-like	*bla*_ADC-25-like_, *bla*_GES-11_, *bla*_OXA-23_, *bla*_OXA-66_, *bla*_TEM-1D_	*aadA2, aadB-*like, *aph(3′)-VIa-*like, *strA-*like, *strB-*like, *aacA4-*like			*dfrA7*	*aac(6′)Ib-cr*-like
WEST-S5-44, *A. radioresistens*, 2013	n.a.	n.a.	n.a.			*bla_OXA-815_*					
Iraq-1, *A. dijkshoorniae*, 2010	1605	1141	n.a.			*bla*_OXA-819_					

## Data Availability

All relevant data are provided in the manuscript, its figure, its table, and its Appendix A. Raw sequence data are deposited and available as stated in the methods chapter. Further raw data can be made available on reasonable request.

## Appendix A

**Table A1:** Results of querying genomes with the TCS function of JSpeciesWS.

Isolate WEST-S5-44								
**Pos.**	**Species**	**Strain**	**Domain**	**Phylum**	**Class**	**Order**	**Family**	**Z-Score**
1	Acinetobacter radioresistens SH164	SH164	Bacteria	Proteobacteria	Gammaproteobacteria	Pseudomonadales	Moraxellaceae	0.99921
2	Acinetobacter radioresistens NIPH 2130	NIPH 2130	Bacteria	Proteobacteria	Gammaproteobacteria	Pseudomonadales	Moraxellaceae	0.99869
3	Acinetobacter radioresistens DSM 6976 = NBRC 102413 = CIP 103788	CIP 103788	Bacteria	Proteobacteria	Gammaproteobacteria	Pseudomonadales	Moraxellaceae	0.99862
4	Acinetobacter radioresistens SK82	SK82	Bacteria	Proteobacteria	Gammaproteobacteria	Pseudomonadales	Moraxellaceae	0.99842
5	Acinetobacter radioresistens WC-A-157	WC-A-157	Bacteria	Proteobacteria	Gammaproteobacteria	Pseudomonadales	Moraxellaceae	0.99811
6	Acinetobacter sp. 1461402	1461402	Bacteria	Proteobacteria	Gammaproteobacteria	Pseudomonadales	Moraxellaceae	0.99798
7	Acinetobacter sp. 230853	230853	Bacteria	Proteobacteria	Gammaproteobacteria	Pseudomonadales	Moraxellaceae	0.99788
8	Acinetobacter sp. 1239920	1239920	Bacteria	Proteobacteria	Gammaproteobacteria	Pseudomonadales	Moraxellaceae	0.99774
9	Acinetobacter sp. 263903-1	263903-1	Bacteria	Proteobacteria	Gammaproteobacteria	Pseudomonadales	Moraxellaceae	0.99763
10	Acinetobacter sp. 272263	272263	Bacteria	Proteobacteria	Gammaproteobacteria	Pseudomonadales	Moraxellaceae	0.99756
Isolate Iraq-1								
**Pos.**	**Species**	**Strain**	**Domain**	**Phylum**	**Class**	**Order**	**Family**	**Z-Score**
1	Acinetobacter lactucae ABBL098	ABBL098	Bacteria	Proteobacteria	Gammaproteobacteria	Pseudomonadales	Moraxellaceae	0.99922
2	Acinetobacter pittii ABBL016	ABBL016	Bacteria	Proteobacteria	Gammaproteobacteria	Pseudomonadales	Moraxellaceae	0.99922
3	Acinetobacter lactucae NRRL B-41902	NRRL B-41902	Bacteria	Proteobacteria	Gammaproteobacteria	Pseudomonadales	Moraxellaceae	0.999
4	Acinetobacter sp. 1542444	1542444	Bacteria	Proteobacteria	Gammaproteobacteria	Pseudomonadales	Moraxellaceae	0.99894
5	Acinetobacter pittii ABBL148	ABBL148	Bacteria	Proteobacteria	Gammaproteobacteria	Pseudomonadales	Moraxellaceae	0.99894
6	Acinetobacter pittii null	null	Bacteria	Proteobacteria	Gammaproteobacteria	Pseudomonadales	Moraxellaceae	0.99892
7	Acinetobacter pittii ABBL126	ABBL126	Bacteria	Proteobacteria	Gammaproteobacteria	Pseudomonadales	Moraxellaceae	0.99891
8	Acinetobacter pittii ABBL019	ABBL019	Bacteria	Proteobacteria	Gammaproteobacteria	Pseudomonadales	Moraxellaceae	0.9989
9	Acinetobacter pittii ABBL033	ABBL033	Bacteria	Proteobacteria	Gammaproteobacteria	Pseudomonadales	Moraxellaceae	0.9989
10	Acinetobacter pittii ANC 4050	ANC 4050	Bacteria	Proteobacteria	Gammaproteobacteria	Pseudomonadales	Moraxellaceae	0.99885

**Table A2:** Phenotypic resistance testing of the *Acinetobacter* spp. isolates based on micro-broth dilution (applied for colistin) and automated resistance testing using a VITEK-II device (BioMéreux, Nürtingen, Germany; applied for all other assessed antimicrobial substances). MICs (minimum inhibitory concentrations) in µg/mL are shown in brackets. Interpretation was performed according to EUCAST NAK (European Committee on Antimicrobial Susceptibility Testing “Nationales Antibiotia-Sensitivitätstest-Kommittee”). S = susceptible. I = Susceptible at increased dosage. R = Resistant. N.d. = not defined.

Strain Number	Species	Imipenem	Meropenem	Amikacin	Gentamicin	Tobramycin	Ciprofloxacin	Cotrimoxazole	Colistin
Iraq-2	*A. baumannii*	R (8)	I (8)	S (≤2)	R (8)	S (4)	R (≥4)	R (160)	S (0.0625)
AFG-1	*A. baumannii*	R (≥16)	R (≥16)	S (≤2)	S (4)	S (2)	R (≥4)	R (≥320)	S (0.5)
AFG-2	*A. baumannii*	R (≥16)	R (≥16)	S (≤2)	S (4)	S (≤1)	R (≥4)	R (≥320)	S (0.25)
AFG-3	*A. baumannii*	R (8)	I (8)	S (8)	S (4)	S (2)	R (≥4)	R (≥320)	S (2)
AFG-4	*A. baumannii*	R (8)	R (≥16)	S (≤2)	S (4)	S (≤1)	R (≥4)	R (≥320)	S (1)
HBG-L1-1	*A. baumannii*	R (≥16)	R (≥16)	S (≤2)	R (≥16)	R (≥16)	R (≥4)	R (≥320)	S (0.5)
HBG-L2-9	*A. baumannii*	R (≥16)	I (8)	S (≤2)	R (8)	R (8)	R (≥4)	R (≥320)	S (0.5)
WEST-S6-47	*A. baumannii*	I (1)	I (4)	R (≥64)	R (8)	R (8)	R (≥4)	R (≥320)	S (1)
WEST-S1-31	*A. baumannii*	R (≥16)	R (≥16)	S (4)	R (≥16)	R (≥16)	R (≥4)	R (≥320)	S (0.5)
WEST-S6-50	*A. baumannii*	R (≥16)	R (≥16)	S (4)	R (≥16)	R (≥16)	R (≥4)	R (≥320)	S (1)
HBG-S4-64	*A. baumannii*	R (≥16)	R (≥16)	S (8)	R (≥16)	R (≥16)	R (≥4)	S (≤20)	S (0.25)
HBG-S1-56	*A. baumannii*	R (≥16)	R (≥16)	S (≤2)	S (4)	S (≤1)	R (≥4)	S (≤20)	S (0.125)
HBG-S7-73	*A. baumannii*	R (≥16)	R (≥16)	S (4)	R (≥16)	R (≥16)	R (≥4)	S (≤20)	S (0.25)
HBG-S2-62	*A. baumannii*	R (≥16)	R (≥16)	R (16)	R (≥16)	R (≥16)	R (≥4)	R (≥320)	S (1)
WEST-S5-44	*A. radioresistens*	S (≤0.25)	S (≤0.25)	S (≤2)	S (≤1)	S (≤1)	I (≤0.25)	S (≤20)	S (0.5)
Iraq-1	*A. dijkshoorniae*	S (≤0.25)	I (4)	S (≤2)	S (≤1)	S (≤1)	I (0.5)	S (≤20)	S (0.125)
